# Behavioral phenotype, intestinal microbiome, and brain neuronal activity of male serotonin transporter knockout mice

**DOI:** 10.1186/s13041-023-01020-2

**Published:** 2023-03-29

**Authors:** Hirotaka Shoji, Kazutaka Ikeda, Tsuyoshi Miyakawa

**Affiliations:** 1 Division of Systems Medical Science, Center for Medical Science, Fujita Health University, Toyoake, Aichi 470-1192 Japan; 2 Addictive Substance Project, Tokyo Metropolitan Institute of Medical Science, 2-1-6 Kamikitazawa, Setagaya-ku, Tokyo, 156-8506 Japan

**Keywords:** Serotonin transporter, Anxiety, Depression, Behavioral test battery, Gut microbiota, c-Fos, Mice

## Abstract

The serotonin transporter (5-HTT) plays a critical role in the regulation of serotonin neurotransmission. Mice genetically deficient in 5-HTT expression have been used to study the physiological functions of 5-HTT in the brain and have been proposed as a potential animal model for neuropsychiatric and neurodevelopmental disorders. Recent studies have provided evidence for a link between the gut-brain axis and mood disorders. However, the effects of 5-HTT deficiency on gut microbiota, brain function, and behavior remain to be fully characterized. Here we investigated the effects of 5-HTT deficiency on different types of behavior, the gut microbiome, and brain c-Fos expression as a marker of neuronal activation in response to the forced swim test for assessing depression-related behavior in male 5-HTT knockout mice. Behavioral analysis using a battery of 16 different tests showed that 5-HTT−/− mice exhibited markedly reduced locomotor activity, decreased pain sensitivity, reduced motor function, increased anxiety-like and depression-related behavior, altered social behavior in novel and familiar environments, normal working memory, enhanced spatial reference memory, and impaired fear memory compared to 5-HTT+/+ mice. 5-HTT+/− mice showed slightly reduced locomotor activity and impaired social behavior compared to 5-HTT+/+ mice. Analysis of 16S rRNA gene amplicons showed that 5-HTT−/− mice had altered gut microbiota abundances, such as a decrease in *Allobaculum*, *Bifidobacterium*, *Clostridium sensu stricto*, and *Turicibacter*, compared to 5-HTT+/+ mice. This study also showed that after exposure to the forced swim test, the number of c-Fos-positive cells was higher in the paraventricular thalamus and lateral hypothalamus and was lower in the prefrontal cortical regions, nucleus accumbens shell, dorsolateral septal nucleus, hippocampal regions, and ventromedial hypothalamus in 5-HTT−/− mice than in 5-HTT+/+ mice. These phenotypes of 5-HTT−/− mice partially recapitulate clinical observations in humans with major depressive disorder. The present findings indicate that 5-HTT-deficient mice serve as a good and valid animal model to study anxiety and depression with altered gut microbial composition and abnormal neuronal activity in the brain, highlighting the importance of 5-HTT in brain function and the mechanisms underlying the regulation of anxiety and depression.

**Supplementary Information:**

The online version contains supplementary material available at 10.1186/s13041-023-01020-2.

## Introduction

The serotonin transporter (5-HTT) is expressed on the presynaptic membrane of serotonergic neurons and reuptakes serotonin (5-HT) from the synaptic cleft into presynaptic terminals to recycle 5-HT for future release, playing a critical role in the regulation of 5-HT neurotransmission [1]. Many studies have suggested that dysregulation of the 5-HT system is associated with neuropsychiatric and neurodevelopmental disorders, such as anxiety and depression, and 5-HTT is a major target for antidepressants and anxiolytics [2–5]. Genetic association studies have reported that genetic variants of 5-HTT, well known as the 5-HTT gene-linked polymorphic region (5-HTTLPR), are associated with increased neuroticism, anxiety, and depression [6–8]. In addition, the short variant of the polymorphism is suggested to reduce the transcriptional efficiency of the 5-HTT gene promoter, resulting in decreased 5-HTT expression and 5-HT uptake [6, 9, 10].

Genetic animal models of 5-HTT deficiency offer promising opportunities to investigate the causal relationship between 5-HTT deficiency and brain function. Earlier and subsequent studies have focused on anxiety and depression phenotypes, reporting that mice genetically deficient in 5-HTT expression exhibited decreased locomotor activity and increased anxiety-like and depression-related behaviors [11–29], which vary with their genetic and environmental background [11, 12, 19, 20]. In contrast, a relatively small number of studies have examined other behavioral domains in 5-HTT knockout (KO) mice, reporting normal sensory function [14, 18], reduced motor performance [11, 14, 18, 19], normal or decreased social behavior [23, 27, 28, 30], and altered memory function [27, 31–33], which await further investigation under various test conditions to confirm the behavioral consequences of 5-HTT deficiency.

The gut microbiota plays an essential role in shaping and modulating the gut-brain axis. There is increasing evidence that the imbalance of the microbiota-gut-brain axis leads to dysregulation of neurotransmission, inflammation, the immune system, and the endocrine system [34, 35], which may be associated with neuropsychiatric and neurodevelopmental disorders, including depression [36–38]. Approximately 95% of the total 5-HT in the body is found in the gastrointestinal tract. 5-HTT mediates 5-HT reuptake in platelets, which allows 5-HT to be distributed to peripheral tissues and contributes to various physiological processes, such as bone remodeling and energy metabolism [39–42]. A recent study reported that 5-HTT−/− mice had higher abundances of *Bacilli* species and lower abundances of *Bifidobacterium* species and *Akkermansia muciniphilia* than 5-HTT+/+ mice in fecal and cecal samples [43]. These findings suggest that altered gut microbial composition via 5-HTT deficiency is associated with changes in physiological and behavioral functions. Various genetic and environmental factors can influence the gut microbial composition [44, 45], and are potential confounding variables that may lead to different outcomes between laboratories. Therefore, further investigation and replication of the association between 5-HTT deficiency and gut microbiota is needed.

Several lines of evidence indicate specific brain neural circuits responsible for emotional and mood regulation [46–48]. In animal studies, c-Fos protein expression has been widely used as a marker of neuronal activity in response to behavioral tests to map functional brain regions associated with anxiety and depression [49–52]. The forced swim test is one of the extensively used behavioral paradigms for screening for new drugs with potential antidepressant effects and assessing depression-related behavior in rodents [53, 54]. In the behavioral test, 5-HTT−/− mice showed increased depression-related behaviors [11, 14]. Brain mapping of c-Fos after exposure to the forced swim test may provide valuable insights into the brain circuits involved in the depressive state induced by 5-HTT deficiency.

There are advantages to using a multifaceted approach to behavioral phenotyping through a battery of behavioral tests, which allows us to characterize various domains of behavior in different test situations, thus minimizing inappropriate interpretation of behavioral data by multiple tests [55–57]. In the present study, to understand the brain function of 5-HTT and to evaluate 5-HTT-deficient mice as an animal model for neuropsychiatric and neurodevelopmental disorders, we first assessed various domains of behavior in male 5-HTT heterozygous KO and homozygous KO mice and their wild-type controls using a battery of behavioral tests, including 16 different tests assessing locomotor activity in novel and familiar environments, sensory and motor function, anxiety-like and depression-related behaviors, social behaviors in novel and familiar environments, prepulse inhibition, working memory, spatial reference memory, contextual and cued fear memory, and remote memory. Although many studies have performed tests for behavioral phenotyping of 5-HTT-deficient mice, some of the behavioral tests and specific behavioral measures (e.g., social interaction test in a novel and familiar environments, some major measures for the acquisition session and probe trial in Barnes maze test, contextual fear conditioning test, and remote memory test) have not been investigated. Thus, our behavioral analysis allows us to reevaluate and further explore the behavioral phenotypes of 5-HTT-deficient mice in different testing situations. Second, we analyzed the fecal microbiota in 5-HTT homozygous KO mice and their wild-type controls to investigate genotype-dependent changes in gut microbial composition in our environment and to explore the potential relationships between 5-HTT deficiency, gut microbiota, and behavior. Finally, to investigate the stress response and brain regions associated with increased depression-related behavior caused by deletion of the 5-HTT gene, plasma corticosterone levels and brain c-Fos expression after the forced swim test were assessed in 5-HTT homozygous KO mice and their wild-type controls.

## Materials and methods

### Animals

5-HTT KO mice were derived from the colony of the originally generated mutants as previously described [13, 15]. After backcrossing to C57BL/6JJcl mice (CLEA Japan, Inc., Tokyo, Japan) for at least ten generations, the mutants were maintained by crossing male heterozygous KO mice and female heterozygous KO mice. In this study, 5-HTT homozygous KO (5-HTT−/−), 5-HTT heterozygous KO (5-HTT+/−), and wild-type (5-HTT+/+) males were obtained from the litters produced by crossing male and female 5-HTT+/− mice in the same manner. Some of the litters used in this study did not contain WT males. Therefore, some WT males used as controls were not littermates of the heterozygous KO mice and/or the homozygous KO mice (for detailed information about the genotype of each mouse in each litter, see “Mouse Phenotype Database”, http://www.mouse-phenotype.org/). After weaning at approximately 4 weeks of age, mice were genotyped and housed in groups (two to four per cage) of the same genotype and sex in plastic cages (250 × 182 × 139 mm) with paper chips for bedding (Paper Clean; Japan SLC, Inc., Shizuoka, Japan). Rooms were maintained on a 12-h light/dark cycle (lights on at 7:00 am) at 23 ± 2 °C. Food (CRF-1, Oriental Yeast Co., Ltd., Tokyo, Japan) and water were provided ad libitum. A total of 33 male 5-HTT homozygous KO (5-HTT−/−), 16 male 5-HTT heterozygous KO (5-HTT+/−), and 36 male wild-type control (5-HTT+/+) mice were used in this study. All experimental procedures were approved by the Institutional Animal Care and Use Committee of Fujita Health University.

### Behavioral tests

Male 5-HTT homozygous KO (5-HTT−/−, n = 15), 5-HTT heterozygous KO (5-HTT+/−, n = 16), and their wild-type control (5-HTT+/+, n = 15) mice, 16–22-weeks (4–5-months) old at the beginning of the experiment, were subjected to a battery of behavioral tests in the following order (see Additional file 4: Table S1): general health and neurological screen, light/dark transition, open field, elevated plus maze, hot plate, social interaction, rotarod, startle response/prepulse inhibition, Porsolt forced swim, three-chamber social approach, T-maze spontaneous alternation, Barnes maze, tail suspension, sucrose preference, contextual and cued fear conditioning, and home cage social interaction tests, as previously described [58]. After each test, the floors and walls of the testing apparatuses were cleaned with 70% ethanol solution and hypochlorous acid water to prevent a bias based on olfactory cues. With the exception of the sucrose preference test, behavioral tests were conducted between 9:00 am and 5:00 pm.

### General health and neurological screen

Physical characteristics, including body weight and rectal temperature, were recorded. Neuromuscular strength was assessed by the grip strength and wire hang tests. Forelimb grip strength was measured using a grip strength meter (O’Hara & Co., Tokyo, Japan). Mice were lifted by their tails to grasp a wire grid with their forelimbs. They were then gently pulled back until they released the grid. The peak force of the grip strength was recorded in Newtons (N). In the wire hang test, mice were placed on a wire mesh (O’Hara & Co., Tokyo, Japan) which was then gently inverted so that the mice grasped the wire. The latency to fall from the wire was recorded with a 60-s cut-off time.

### Light/dark transition test

The light/dark transition test, developed by Crawley and colleagues [59] to assess anxiety-like behavior, was performed as previously described [60]. The apparatus consisted of a cage (21 × 42 × 25 cm) divided into two equal chambers by a partition with a door (O’Hara & Co., Tokyo, Japan). One chamber had white plastic walls and was brightly lit (390 lx) by lights mounted above the ceiling of the chamber. The other chamber had black plastic walls and was dark (2 lx). Both chambers had a white plastic floor. Mice were placed in the dark chamber and allowed to move freely between the two chambers for 10 min with the door open. Distance traveled (cm), the number of transitions, latency to enter the light chamber (s), and time spent in the light chamber (s) were automatically recorded using the ImageLD software (see “Image analysis”).

### Open field test

The open field test was conducted to assess anxiety-like behavior, habituation to a novel environment, and locomotor activity [61–64] by measuring behaviors for 120 min in the open field apparatus with the VersaMax activity monitoring system (Accuscan Instruments, Columbus, OH, USA). The open field arena was made of acrylic with transparent walls and a white floor (40 × 40 × 30 cm). The floor of the central area, defined as 20 cm × 20 cm, was illuminated at 100 lx. Each mouse was placed in one corner of an open field and allowed to explore freely for 120 min. Distance traveled (cm), vertical activity (rearing measured by counting the number of photobeam interruptions), time spent in the center area (s), and stereotypic counts (beam-break counts for stereotyped behaviors) were measured in each 5-min block.

### Elevated plus maze test

The elevated plus maze test, which is widely used to assess anxiety [65], was performed as previously described [66, 67]. The apparatus consisted of two open arms (25 × 5 cm) and two closed arms of the same size with 15-cm-high transparent walls and a central square (5 × 5 cm) connecting the arms (O’Hara & Co., Tokyo, Japan). The floor of the apparatus was made of white plastic plates and was elevated to 55 cm above the floor. The open arms were surrounded by a raised ledge (3-mm thick and 3-mm high) to prevent mice from falling off the arms. Arms of the same type were located opposite one another. The illumination level at the central area was 100 lx. Each mouse was placed in the central square of the maze facing one of the closed arms. The number of arm entries, distance traveled (cm), percentage of entries into open arms, and percentage of time spent in open arms were measured during a 10-min test period. Data acquisition and analysis were performed automatically using the ImageEP software.

### Hot plate test

The hot plate test was used to assess sensitivity to a painful stimulus. Each mouse was placed on a hot plate (55.0 ± 0.1 °C; Columbus Instruments, Columbus, OH, USA), and the latency to the first paw response (s) was recorded with a cut-off time of 15 s. The paw response was defined as either a paw lick or a foot shake.

### Social interaction test

The social interaction test was used to assess social behavior in a novel environment. Weight-matched mice (mean ± SD for differences in body weight: 5-HTT+/+, 2.15 ± 1.32 g; 5-HTT+/−, 1.87 ± 0.97 g; 5-HTT−/−, 1.43 ± 1.34 g) of the same genotype, housed in different cages, were placed together in a white plastic box (40 × 40 × 30 cm) and allowed to explore freely for 10 min. The central area of the box was illuminated at 100 lx. The total number of contacts, total duration of contacts (s), total duration of active contacts (s), mean duration per contact (s), and total distance traveled (cm) were measured automatically using the ImageSI software. The active contact was measured when the two mice made contact and one or both mice moved with a velocity of at least 10 cm/s.

### Rotarod test

Motor coordination and balance were assessed using the rotarod test. Mice were placed on a rotating drum (3 cm diameter, UGO Basile Accelerating Rotarod). They were given three trials per day for two consecutive days. The latency to fall off the rod (s) was measured. The speed of the rotarod accelerated from 4 to 40 rpm over 300 s.

### Three-chamber social approach test

The apparatus consisted of a rectangular, three-chambered box and a lid with a video camera (O’Hara & Co., Tokyo, Japan). Each chamber was made of white plastic (20 × 40 × 47 cm) and the partitions were made of transparent acrylic with a small square opening (5 × 3 cm). The three-chamber test was conducted as previously described [68]. First, each test mouse was placed in the center chamber of the apparatus, in which empty wire cages (9 cm in diameter, 11 cm in height, with vertical bars 0.5 cm apart) were placed in the corners of each side chamber, and was allowed to explore for 10 min (habituation session). Next, an unfamiliar C57BL/6 J male mouse (stranger 1; 9–10 weeks old; strangers were obtained from Charles River Laboratories Japan, Inc., Kanagawa, Japan) that had no prior contact with the test mice was placed in the wire cage located in one of the side chambers. The location of the stranger mouse in the left or right chamber was systematically alternated between trials. The test mouse was placed in the center chamber and allowed to explore for a 10-min session to assess sociability (sociability test). Then, a second stranger mouse (stranger 2; 9–10 weeks old) was placed in the wire cage that had been empty during the first 10-min session to assess social preference for the new stranger (social novelty preference test). Thus, the test mouse had a choice between the first, already-investigated, now-familiar mouse (stranger 1) and the new, unfamiliar mouse (stranger 2). Time spent in each chamber and around each cage was automatically calculated from video images using the ImageCSI software.

### Acoustic startle response/prepulse inhibition test

Startle response and prepulse inhibition tests were performed using a startle reflex measurement system (O’Hara & Co., Tokyo, Japan). Mice were placed in a Plexiglas cylinder and left undisturbed for 10 min. A loud sound stimulus (110 or 120 dB, white noise, 40 ms) was presented as a startle stimulus. A prepulse sound (74 or 78 dB, white noise, 20 ms) was presented 100 ms before the startle stimulus to assess prepulse inhibition. A test session consisted of six trial types (i.e., two types of startle stimulus-only trials, and four types of prepulse inhibition trials: 74–110, 78–110, 74–120, and 78–120 dB). Six blocks of the six trial types were presented in a pseudo-random order such that each trial type was presented once within a block. The average interval was 15 s (range: 10–20 s). A 70-dB white noise was presented as a background noise during the test. The peak amplitude of the startle responses to the stimuli was recorded for 400 ms from the onset of the prepulse stimulus. The percent PPI was calculated for each mouse using the following formula: percent PPI = 100 × [1 − (startle response amplitude in prepulse + startle trial)/(startle response amplitude in startle stimulus-only trial)].

### Porsolt forced swim test

The Porsolt forced swim test, developed by Porsolt and colleagues [53], was used to assess depression-related behavior. Mice were placed in a clear plastic cylinder (20 cm height × 10 cm diameter, O’Hara & Co., Tokyo, Japan) filled with water (approximately 21 °C) to a depth of 8 cm for 10 min. The percentage of immobility time was automatically calculated using the ImagePS/TS software as previously described [58, 69].

### T-maze spontaneous alternation test

The T-maze spontaneous alternation test was conducted to assess spatial working memory using a modified automatic T-maze apparatus (O'Hara & Co.), as previously described [58, 70]. The apparatus consisted of white plastic runways with 25-cm-high walls. It was partitioned into six areas: the stem of the T, a straight runway, left and right arms, and the passageways connecting the arms to the stem of the T. Mice were subjected to a session of 10 trials per day for two days (a cut-off time of 50 min). Each trial consisted of a forced-choice followed by a free choice (inter-trial interval, 60 s). In the forced-choice trial, mice were forced to enter either the left or right arm of the T-maze and were held in the arm for 10 s. After the 10-s period, the doors of the passageway connecting the arm to the stem of the T were opened and the mouse was allowed to return to the start compartment. Three seconds after the mice entered the start compartment, a free-choice trial began. The mice were allowed to choose one of the arms in the free-choice trial, and if the mice entered the arm opposite to the arm they entered in the forced-choice trial, the response was recorded as a correct response. The mean percentage of correct responses across two sessions was calculated. Data acquisition and analysis were performed automatically using the ImageTM software (see "Image analysis").

### Barnes maze test

The Barnes circular maze test [71] was used to test spatial reference memory on ‘dry land’, a white circular surface 1.0 m in diameter with 12 holes equally spaced around the perimeter (O’Hara & Co., Tokyo, Japan). The circular open field was elevated 75 cm above the floor. A black Plexiglas escape box (17 × 13 × 7 cm) was placed under one of the holes (target hole). The acquisition session consisted of two trials per day for nine days. In each trial, a mouse was placed in the center of the maze and allowed to explore. If a mouse did not enter the escape box within a maximum of 5 min, it was gently guided to the escape hole. After entering the escape hole, the mouse remained in the escape box for 30 s before returning to the holding cage. The location of the target was constant for a given mouse, but randomized across mice. The maze was rotated daily, but the spatial location of the target was kept constant for the distal visual cues to avoid bias due to an olfactory cue or proximal cues. The latency to reach the target hole (s), the number of errors to reach the target hole, and the distance traveled first to reach the target hole (cm) were automatically recorded by the ImageBM software. One day and 28 days after the last acquisition session, probe trials without the escape box were performed to assess spatial reference memory. In the probe test, the time spent around each hole (s) was measured using the ImageBM software.

### Tail suspension test

The tail suspension test was performed to evaluate depression-related behavior [72]. Mice were suspended 30 cm above the floor in a visually isolated area using adhesive tape placed approximately 1 cm from the tip of the tail. Immobility time was measured for a 10-min test period using the ImagePS/TS software in the same manner as for the forced swim test.

### Sucrose preference test

After the tail suspension test, mice were individually housed in plastic cages (250 × 182 × 139 mm) with fresh paper chips as bedding and provided with two bottles of filtered tap water. After one day of acclimation to the housing conditions, mice were given one bottle of water and a second bottle of 1% sucrose solution, with the left/right position counterbalanced across genotypes of animals. The bottles were weighed at approximately 24-h intervals to measure water and sucrose intake on each day of the four-day session, with the left–right position changed daily. Sucrose preference was expressed as 100 × [(sucrose intake in grams)/(sucrose intake in grams + water intake in grams)].

### Contextual and cued fear conditioning test

The contextual and cued fear conditioning test was conducted to assess fear memory using an automated video analysis system [73]. First, mice were placed in a conditioning chamber (26 × 34 × 29 cm) in a sound-attenuated room and allowed to explore freely for 2 min. Next, the animals were presented with an auditory cue (55 dB white noise) that served as a conditioned stimulus (CS) for 30 s. During the last 2 s of the CS, the mice received a mild footshock (0.3 mA, 2 s) as an unconditioned stimulus (US). Two more CS-US pairings were then presented at 120-s intervals. One day and 29 days after the conditioning session, a context test was performed in the conditioning chamber. More than 3 h after the context test, a cued test in an altered context was performed in a triangular box (35 × 35 × 40 cm) made of opaque white plastic in another sound-attenuated room. In the cued test, after the initial 3-min period without CS presentation, the CS was presented during the last 3-min period. In each test, video images were recorded at one frame per second. Freezing time (%) and distance traveled (cm) in each trial were measured automatically from the images using the ImageFZ software, as previously described [73]. Images were also recorded at a rate of 4 frames per second for 6 s from 2 s before delivery of a 2 s footshock until 2 s after the footshock, and distance traveled (cm) was measured as an index of footshock sensitivity.

### Home cage social interaction test

The home cage social interaction test was conducted to assess social behavior and activity levels under familiar conditions in a home cage. This test was performed 11 days after the context and cued tests on day 2 of the fear conditioning test (the second session of context and cued tests, 29 days after the conditioning, was performed seven days after the home cage social interaction test). The social interaction monitoring system consisted of a home cage with paper chips for bedding and a cage top with an infrared video camera (25 × 15 × 23.5 cm, internal dimensions). Two mice of the same genotype housed in separate cages were placed together in the cage. Video images were recorded at a rate of 1 frame per second. Social interaction was measured by automatically counting the number of animals detected in each frame using the ImageHA software. The activity level of animals was quantified by measuring the number of pixels that changed between each pair of consecutive images. The mean number of animals and total activity level in each 1-h bin were calculated for 1 week.

### Image analysis for behavioral test

Image analysis software (ImageLD/EP/SI/CSI/PS/TS/TM/BM/FZ/HA) were used to automatically analyze mouse behaviors, as previously described [60, 66, 70, 73]. The software, based on the public domain ImageJ program (developed by Wayne Rasband at the National Institute of Mental Health, Bethesda), were developed and modified for each test by Tsuyoshi Miyakawa. The ImageLD/EP/TM/FZ programs can be freely downloaded from the “Mouse Phenotype Database” (http://www.mouse-phenotype.org/).

### Fecal sample collection, 16S rRNA gene sequencing, and data analysis

Fecal samples were collected from an independent group of naïve male 5-HTT−/− and 5-HTT+/+ mice (n = 8, each genotype; two mice per cage were used; in each genotype), which were individually placed in an empty cage (250 × 182 × 139 mm) precleaned with 65% ethanol. The feces were quickly collected in a tube and stored at − 80 °C. DNA from each fecal sample was extracted by a bead-based method, as previously performed [74]. Prokaryote universal primers (Pro341F and Pro805R) with the sample-specific 8-bp dual-index barcode sequences were used to amplify V3 and V4 regions of 16S rDNA genes by polymerase chain reaction [74, 75]. The barcoded amplicons were paired-end sequenced on the Illumina MiSeq platform using the MiSeq reagent kit v3 (600 cycles, 2 × 284-bp cycle; Illumina, San Diego, CA, USA). Paired-end reads were joined using the fastq-join program [76]. The joined reads with a quality value score ≥ 20 for > 99% of the sequence were extracted using FASTX-Toolkit [77] and were used for further analysis. The chimeric sequences were removed using uSearch61 software [78, 79]. Taxonomy assignment from the sequence reads was performed using Metagenome@KIN software (World Fusion, Tokyo, Japan) and the database RDP MultiClassifier ver.2.11 [80] with an 80% confidence level. The amplicon sequencing and taxonomic assignment were performed by TechnoSuruga Laboratory Co., Ltd. (Shizuoka, Japan).

The read counts were analyzed to assess α-diversity (species richness and evenness from the rarefied counts; observed number of microbial genera, Chao1 index, Shannon index, and Simpson index) using R packages 'vegan' ver. 2.5–7 [81]. In addition, β-diversity was visualized with principal coordinate analysis (PCoA) with Bray–Curtis dissimilarity, and permutational multivariate analysis of variance (PERMANOVA) was used to assess microbial differences using the adonis function in the R package 'vegan'. The relative abundance of microbiota at the genus level was analyzed using the linear discriminant analysis (LDA) effect size (LEfSe) method [82] to identify taxonomic features characterizing the differences between the genotypes (LDA score > 3, p < 0.05).

### Plasma corticosterone measurement

Blood samples were collected from the facial vein or submandibular vein of an independent group of naïve male 5-HTT−/− (n = 5) and 5-HTT+/+ (n = 6) mice using a Goldenrod Animal Lancet (MEDIpoint, Inc., NY, USA) to measure basal plasma corticosterone levels during the light phase (11:30–12:30). Samples were placed in tubes containing 1 unit of sodium heparin (Wako Pure Chemical Industries Ltd., Osaka, Japan) and centrifuged at 3000×*g* for 10 min at 4 °C. Supernatants were collected and stored at − 80 °C until measurement. Two days after the first blood collection, mice were subjected to the forced swim test for 10 min (11:00–12:30). Blood samples were collected immediately and 90 min after the test to assess stress-induced corticosterone levels and their time-dependent decline after stress exposure. Plasma corticosterone (CORT) concentrations were determined using an enzyme immunoassay kit (Assay Designs Inc., MI, USA) according to the manufacturer’s protocol.

### c-Fos immunohistochemistry

Immediately after the blood sampling 90 min after exposure to the forced swim test, 5-HTT−/− (n = 5) and 5-HTT+/+ (n = 6) mice were deeply anesthetized and transcardially perfused with saline followed by 4% paraformaldehyde in 0.1 M phosphate buffer (PB). Mice not subjected to the forced swim test and blood sampling as non-exposed groups (5-HTT−/−, n = 5; 5-HTT+/+, n = 7) were treated identically immediately after removal from their home cages. Brain samples were removed and fixed in the same fixative overnight at 4 °C. After the post-fixation, the brains were soaked in 30% sucrose in phosphate buffer saline (PBS) at 4 °C for at least 3 days, then embedded in Tissue-Tek OCT compound (Sakura Finetek Japan Co., Ltd., Tokyo, Japan) under liquid nitrogen, and stored at − 80 °C. Brains were cut into 30-μm-thick coronal sections on a cryostat (CM1850; Leica Biosystems, Wetzlar, Germany). Sections were stored at − 20 °C in a cryoprotectant solution (25% glycerol, 25% ethylene glycol in 0.1 M PB) until use. Floating sections were washed with PBS and incubated in 0.3% H2O2 for 20 min. After washing with PBS, the sections were soaked in normal horse serum (S-2000; Vector Laboratories, CA, USA) in PBS containing 0.3% Triton X-100 for 30 min at room temperature and then were incubated overnight at 4 °C with goat polyclonal anti-c-Fos antibody (1:1000, sc-52G; Santa Cruz Biotechnology, CA, USA). The sections were then washed with PBS and incubated with biotinylated horse anti-goat secondary antibody (1:500, BA-9500; Vector Laboratories, CA, USA) for 30 min, followed by incubation with reagents from the VECTASTAIN Elite ABC kit (PK-6100; Vector Laboratories, CA, USA) for 30 min. Sections were immersed in DAB peroxidase substrate solution (ImmPACT, SK-4105; Vector Laboratories, CA, USA) for 2 min. Sections were then mounted on glass slides, dehydrated in ethanol, cleared in xylene, and coverslipped with Multi Mount 480 (Matsunami Glass Ind., Ltd., Osaka, Japan). Images of the stained sections were collected using a light microscope (BZ-9000; Keyence, Osaka, Japan). At least six sections per animal were used for image analysis of each brain region. The number of c-Fos-positive cells was quantified in 200 μm × 200 μm of brain regions bilaterally (see Fig. 6b). Anatomical localization of brain regions was aided by the use of the illustrations in a stereotaxic atlas ([83]; see Fig. 6b and Additional file 4: Table S5). Cells were counted manually in ImageJ in a blinded fashion.

### Statistical analysis

Statistical analyses were performed using SAS Studio (SAS OnDemand for Academics; SAS Institute, Cary, NC, USA) and R (version 3.6.3). Behavioral data were analyzed by one-way ANOVAs with genotype as the between-subject variable or two-way repeated measures ANOVAs with genotype as between-subject variables and time/trial/block as the within-subject variable. When a genotype × time interaction was significant, simple main effects were analyzed to examine the effect of genotype at each time point. Comparisons of microbial composition between genotypes were performed using Wilcoxon rank sum test. Plasma corticosterone levels were analyzed using two-way repeated measures ANOVAs with genotype and phase (before exposure to the forced swim test, immediately after the test, and 90 min after the test). Brain c-Fos expression was analyzed using a two-way ANOVA with genotype and exposure (no exposure and forced swim exposure). Values in graphs are expressed as the mean ± SEM. Uncorrected p-values are shown.

## Results

### Physical characteristics and neurological functions in 5-HTT-deficient mice

The statistical results of behavioral data of 5-HTT−/−, 5-HTT+/−, and 5-HTT+/+ mice subjected to a behavioral test battery are summarized in Additional file 4: Table S2.

**Fig. 1:**
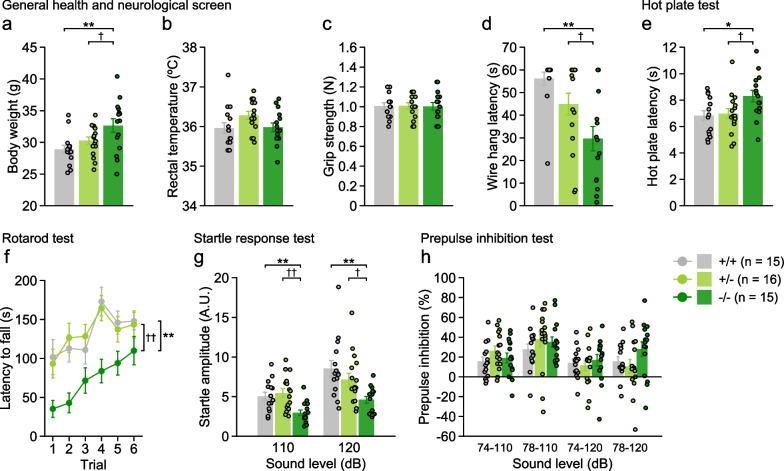
General health and sensory and motor functions in 5-HTT-deficient mice. **a** Body weight (g), **b** body temperature (°C), **c** grip strength (Newton, N), **d** wire hang latency (s), **e** latency to paw lick or foot shake (s) in the hot plate test, **f** latency to fall off a rotating rod (s) in the rotarod test, **g** acoustic startle response to sound stimuli (110 and 120 dB white noise), and **h** prepulse inhibition (%) of the startle response with 74 and 78 dB prepulse stimuli. Values are means ± SEM. Asterisks and daggers indicate statistically significant differences between groups (5-HTT−/− vs. 5-HTT+/+, *p < 0.05 and **p < 0.01; 5-HTT−/− vs. 5-HTT+/−, ^†^p < 0.05 and ^††^p < 0.01)

There were significant effects of genotype on the body weight (Fig. 1a), wire hang latency (Fig. 1d), hot plate latency (Fig. 1e), rotarod latency (Fig. 1f), and acoustic startle responses to 110 dB and 120 dB stimuli (Fig. 1g). No significant effects of genotype were found on the rectal temperature (Fig. 1b), grip strength (Fig. 1c), and prepulse inhibition in any of the trial types (Fig. 1h). Post hoc analysis revealed that 5-HTT−/− mice were significantly heavier than 5-HTT+/+ and 5-HTT+/− mice (Fig. 1a; vs. +/+, p = 0.0017; vs. +/−, p = 0.0359), while body weights were not significantly different between 5-HTT+/+ and 5-HTT+/− mice (p = 0.2238). In the wire hand test (Fig. 1d), 5-HTT−/− mice exhibited a shorter latency to fall off the wire, which is indicative of reduced muscular strength, than 5-HTT+/+ mice (p = 0.0002), and the wire hang latency of 5-HTT+/− mice was intermediate between that of the other two genotypes (vs. +/+, p = 0.0804; vs. −/−, p = 0.0206). The hot plate latency was higher in 5-HTT−/− mice than in 5-HTT+/+ and 5-HTT+/− mice (Fig. 1e; vs. +/+, p = 0.0122; vs. +/−, p = 0.0223), and there were no significant differences between 5-HTT+/+ and 5-HTT+/− mice (p = 0.7733), suggesting reduced pain sensitivity in 5-HTT−/− mice. In the rotarod test (Fig. 1f), 5-HTT−/− mice spent less time on the rotating rod compared with other genotypes, suggesting decreased motor function (vs. +/+, p = 0.0037; vs. +/−, p = 0.0032), while no difference was found between 5-HTT+/− and 5-HTT+/+ mice (p = 0.9919). Acoustic startle responses were decreased in 5-HTT−/− mice compared to other genotypes in response to 110 dB stimulus (vs. +/+, p = 0.0038; vs.  +/−, p = 0.0006) and 120 dB stimulus (vs. +/+, p = 0.0009; vs. +/−, p = 0.0236). There were no significant differences in the startle responses between 5-HTT+/− and 5-HTT+/+ mice (110 dB, p = 0.5560; 120 dB, p = 0.2095).

### Decreased locomotor activity and increased anxiety-like behavior in 5-HTT-deficient mice

**Fig. 2:**
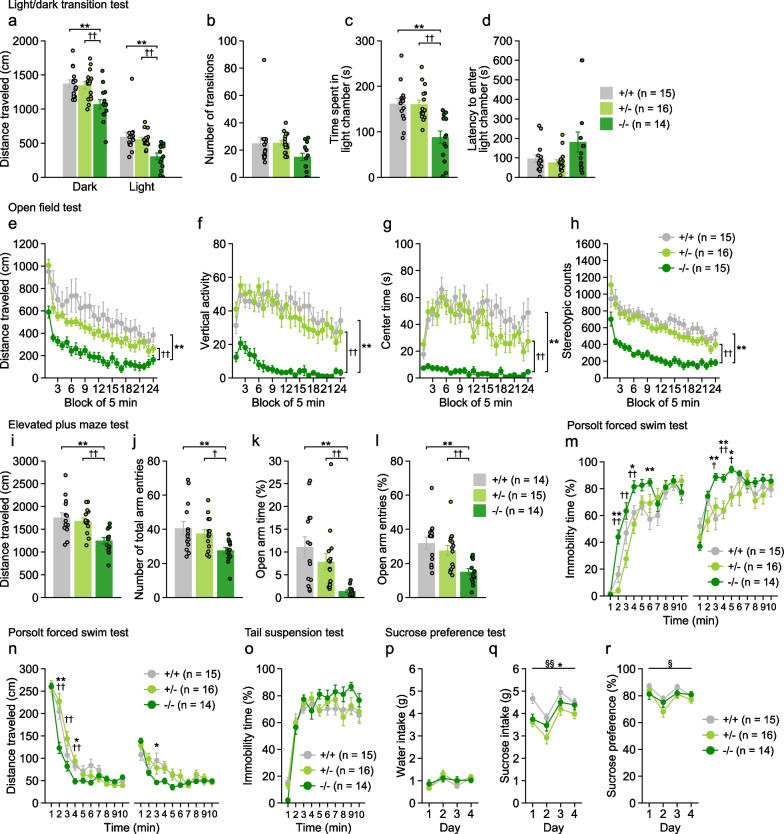
Anxiety-like and depression-related behaviors in 5-HTT-deficient mice. **a**–**d** Light/dark transition test: **a** distance traveled (cm) in the dark and light chambers, **b** number of transitions, **c** time spent in the light chamber (s), and **d** latency to enter the light chamber (s). **e**–**h** Open field test: **e** distance traveled (cm), **f** vertical activity, **g** center time (s), and **h** stereotypic counts for each 5-min block of testing. **i**–**l** Elevated plus maze test: **i** distance traveled (cm), **j** number of arm entries, **k** time spent in open arms (%), and **l** entries into open arms (%). **m**, **n** Porsolt forced swim test: **m** immobility time (%) and **n** distance traveled (cm) for each 1 min-block. **o** Immobility time (%) for each 1-min block in the tail suspension test. (p–r) Sucrose preference test: **p** water intake and **q** 1% sucrose intake, and **r** sucrose preference (%). Values are means ± SEM. Asterisks and daggers indicate statistically significant differences between groups (5-HTT−/− vs. 5-HTT+/+, *p < 0.05 and **p < 0.01; 5-HTT−/− vs. 5-HTT+/−, ^†^p < 0.05 and ^††^p < 0.01; 5-HTT+/− vs. 5-HTT+/+, ^§^ p < 0.05 and ^§§^p < 0.01)

In the light/dark transition test, 5-HTT−/− mice traveled shorter distances in the dark chamber (Fig. 2a: −/− vs. +/+, p = 0.0007; −/− vs. +/−, p = 0.0015) and light chamber (Fig. 2a: −/− vs. +/+, p = 0.0002; −/− vs. +/−, p = 0.0011) and spent less time in the light chamber (Fig. 2c: −/− vs. +/+, p < 0.0001; −/− vs. +/−, p < 0.0001) than the other genotypes of mice. These behaviors were not significantly different between 5-HTT+/− and 5-HTT+/+ mice. There were no significant effects of genotype on the number of transitions (Fig. 2b) and the latency to enter the light chamber (Fig. 2d). These observations indicate that 5-HTT−/− mice showed reduced locomotor activity and increased anxiety-like behavior compared to 5-HTT+/+ and 5-HTT+/− mice.

In the open field test, there were significant main effects of genotype and significant genotype × time interactions on the distance traveled, vertical activity, center time, and stereotypic counts (Fig. 2e–h). For these behavioral measures, significant simple main effects of genotype were found in every 5-min block except for the 23rd block for the distance traveled (all p < 0.05). In almost all 5-min blocks, 5-HTT−/− mice exhibited significantly shorter distance traveled, less vertical activity, and fewer stereotypic behaviors than 5-HTT+/+ and 5-HTT+/− mice (p < 0.05), indicating that 5-HTT−/− mice had reduced locomotor activity. 5-HTT−/− mice also spent significantly less time in the center area, suggestive of increased anxiety-like behavior, than 5-HTT+/+ and 5-HTT+/− mice (p < 0.05). 5-HTT+/− mice showed slightly decreased distance traveled in the 10th and 17th blocks, shorter center time in the 17th and 23rd blocks, and fewer stereotypic counts in the 10th, 14th, 16th, 17th, 19th, 20th, and 23rd blocks than 5-HTT+/+ mice (all p < 0.05).

In the elevated plus maze test, there were significant genotype effects on distance traveled, number of total arm entries, percentage of open arm entries, and percentage of time on open arms (Fig. 2i–l). 5-HTT−/− mice exhibited decreased distance traveled, fewer total arm entries, a lower percentage of open arm entries, and a lower percentage of time on open arms than 5-HTT+/+ and 5-HTT+/− mice (all p < 0.05). There were no significant differences in these behavioral measures between 5-HTT+/− and 5-HTT+/+ mice.

Taken together, these data obtained from three different types of behavioral tests indicate that 5-HTT−/− mice showed reduced locomotor activity and increased anxiety-like behavior compared to 5-HTT+/− and 5-HTT+/+ mice.

### Increased depression-related behavior in 5-HTT-deficient mice

In the forced swim test, significant main effects of genotype and significant genotype × time interactions were found on the percentage of immobility time and distance traveled on days 1 and 2 (Fig. 2m, n). On day 1, 5-HTT−/− mice displayed increased immobility compared to 5-HTT+/+ mice (2nd, 4th, and 6th time blocks, all p < 0.05) and 5-HTT+/− mice (2nd, 3rd, and 4th time blocks, all p < 0.05), while 5-HTT−/− mice traveled shorter distances than 5-HTT+/+ mice (2n and 4th time blocks, all p < 0.05) and 5-HTT+/− mice (2nd, 3rd, and 4th time blocks, all p < 0.05). 5-HTT+/− mice did not differ from 5-HTT+/+ mice in immobility and distance traveled in any time blocks. Similarly, on day 2, 5-HTT−/− mice showed increased immobility compared to 5-HTT+/+ mice (3rd, 4th, and 5th time blocks, all p < 0.05) and 5-HTT+/− mice (3rd, 4th, and 5th time blocks, all p < 0.05), while 5-HTT−/− mice traveled shorter distances than 5-HTT+/+ mice in the 3rd time block (p = 0.0122). There were no significant differences in immobility and distance traveled between 5-HTT+/+ and 5-HTT+/− mice. These data suggest that 5-HTT−/− mice showed increased depression-related behavior than other genotypes of mice.

In the tail suspension test, there was no significant main effect of genotype and no significant genotype × time interaction on the immobility (Fig. 2o).

In the sucrose preference test, there was no significant main effect of genotype and no significant genotype × session interaction on water intake (Fig. 2p). There was a significant main effect of genotype was found on sucrose intake (Fig. 2q: F_2,42_ = 7.04, p = 0.0023) and percent sucrose preference (Fig. 2r; F_2,42_ = 3.60, p = 0.0362). There were no significant genotype × session interactions on sucrose intake and percent sucrose preference, indicating that the genotype effects were not dependent on the test session (Fig. 2q, r). These data suggest that mice of each genotype did not show avoidance of the sucrose solution from test day 1 and that there was no apparent genotype difference in the process of the habituation to the sucrose solution. Post hoc analysis showed that 5-HTT−/− and/or 5-HTT+/− mice had reduced sucrose intake (−/− vs. +/+, p = 0.0471; +/− vs. +/+, p = 0.0005) and sucrose preference (−/− vs. +/+, p = 0.1530; +/− vs. +/+, p = 0.0105) compared to 5-HTT+/+ mice.

### Altered social behavior in 5-HTT-deficient mice

**Fig. 3:**
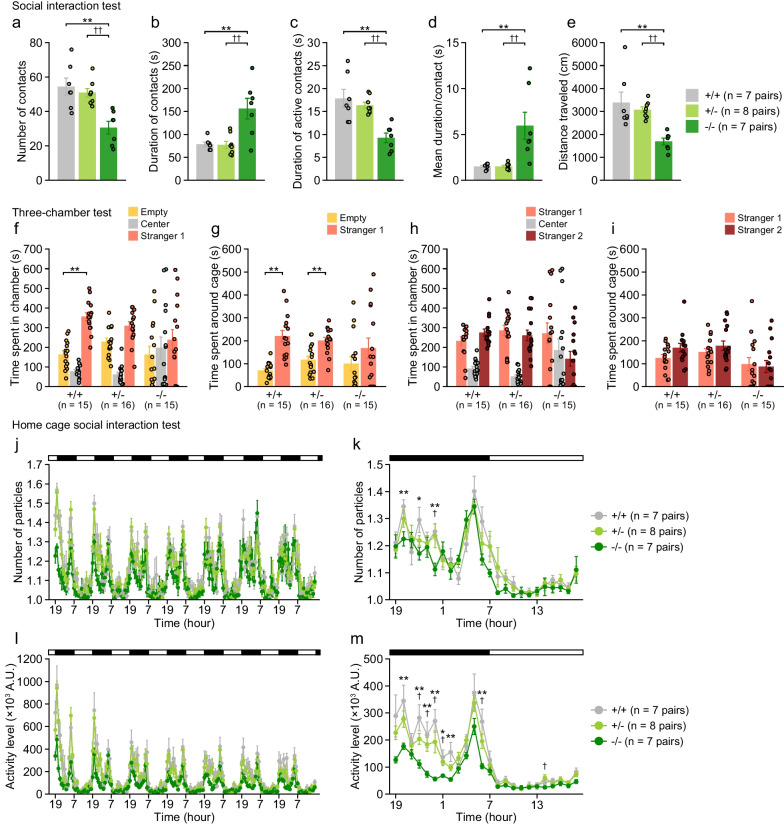
Social behavior in 5-HTT-deficient mice. **a**–**e** Social interaction test in a novel environment: **a** the number of contacts, **b** total duration of contacts (s), **c** total duration of active contacts (s), **d** mean duration per contact (s), and distance traveled (cm). **f**–**i** Three-chamber social approach test: **f** time spent in the chamber with an empty cage, the center chamber, and the chamber with a cage containing a stranger mouse (stranger 1). **g** Time spent around the empty cage and the cage with stranger 1. **h** Time spent in the chamber with the cage containing stranger 1, the center chamber, and the chamber with a cage containing a novel unfamiliar mouse (stranger 2). **i** Time spent around the cage containing stranger 1 and the cage containing stranger 2. **j**–**m** Home cage social interaction test: **j** mean number of particles calculated for each 1-h period over 7 days, **k** mean number of particles averaged over the last 3 days, **l** mean activity level (arbitrary unit, A.U.) for each 1-h period over 7 days, and **m** mean activity level (A.U.) averaged over the last 3 days. Values are means ± SEM. **a**–**e**, **j**–**m** Asterisks and daggers indicate statistically significant differences between groups (5-HTT−/− vs. 5-HTT+/+, *p < 0.05 and **p < 0.01; 5-HTT−/− vs. 5-HTT+/−, ^†^p < 0.05 and ^††^p < 0.01). **f**–**i** Asterisks represent statistical significance with paired t tests (**p < 0.01)

In the one-chamber social interaction test in a novel environment, there were significant effects of genotype on all the behavioral measures (Fig. 3a–e). Compared to 5-HTT+/+ and 5-HTT+/− mice, 5-HTT−/− mice exhibited fewer number of contacts (vs. +/+, p = 0.0003; vs. +/−, p = 0.0010), longer time of contacts (vs. +/+, p = 0.0008; vs. +/−, p = 0.0005), shorter time of active contacts (vs. +/+, p = 0.0002; vs. +/−, p = 0.0009), longer mean duration per contact (vs. +/+, p = 0.0013; vs. +/−, p = 0.0010), and shorter distance traveled (vs. +/+, p = 0.0004; vs. +/−, p = 0.0020). No significant differences in these measures were observed between 5-HTT+/+ and 5-HTT+/− mice.

In the three-chamber sociability test, 5-HTT−/− mice showed no significant differences between time spent in the chamber with stranger 1 and time spent in the chamber with an empty cage (Fig. 3f; t_14_ = 0.97, p = 0.3488) and between time spent around the cage containing stranger 1 and time spent around the empty cage (Fig. 3g; t_14_ = 1.16, p = 0.2663). Although 5-HTT+/− mice spent more time around the cage with stranger 1 than time around the empty cage (Fig. 3g; t_15_ = 3.33, p = 0.0045), they showed no significant difference between time spent in the chamber with stranger 1 and time spent in the chamber with the empty cage (Fig. 3f; t_15_ = 2.09, p = 0.0536). In contrast, 5-HTT+/+ mice spent more time in the chamber with stranger 1 than in the chamber with empty cage (t_14_ = 5.05, p = 0.0002) and spent more time around the cage containing stranger 1 than around the empty cage (t_14_ = 5.24, p = 0.0001). In the social novelty preference test, although there were no significant differences between the time spent in the side chambers with stranger 1 and stranger 2 and between the time spent around the cages containing stranger 1 and stranger 2 (Fig. 3h, i), 5-HTT+/+ mice showed a tendency toward increased time spent around the cage containing stranger 2 compared to the cage containing stranger 1 (Fig. 3i; p = 0.0700). The results of the sociability test and the one-chamber social interaction test indicate the reduced social behavior in 5-HTT−/− mice.

In the home cage social interaction test, pairs of mice of identical genotypes were housed in a cage for seven days. There were significant main effects of genotype and genotype × time interactions on the number of particles (one particle indicates contact between the two mice, and two particles indicate that the mice are not in contact with each other; Fig. 3j) and activity levels (Fig. 3l). For the total period, the number of particles was lower in 5-HTT−/− mice than in other genotypes (vs. 5-HTT+/+, p = 0.0028; vs. 5-HTT+/−, p = 0.0073), suggesting increased physical contact with each other in 5-HTT−/− mice, and activity levels were lower in 5-HTT−/− mice than in other genotypes (vs. 5-HTT+/+, p = 0.0011; vs. 5-HTT+/−, p = 0.0061). Overall, there were no significant differences in these behavioral measures between 5-HTT+/+ and 5-HTT+/− mice. Behavioral data averaged over the last three days were analyzed to assess social behavior and locomotor activity in the familiar condition. Two-way repeated measures ANOVAs revealed that there was a significant genotype × time interaction on the number of particles (Fig. 3k), and there was a significant main effect of genotype and a significant genotype × time interaction on activity levels (Fig. 3m). A lower number of particles was observed in 5-HTT−/− mice than in 5-HTT+/+ mice during the dark phase (8 pm, 10 pm, and 0 am; all p < 0.05) and in 5-HTT+/− mice at 0 am (p = 0.0186). 5-HTT−/− mice showed lower activity levels than other genotypes during the dark phase (all p < 0.05, for 8 pm, 10 pm to 2 am, 6 am, 2 pm except for the case of comparison between 5-HTT−/− and 5-HTT+/+ mice at 2 pm and between 5-HTT−/− and 5-HTT+/− mice at 8 pm and 2 am). No significant differences in behavioral data averaged over the last three days were found between 5-HTT+/+ and 5-HTT+/− mice.

### Normal working memory and altered spatial memory in 5-HTT-deficient mice

**Fig. 4:**
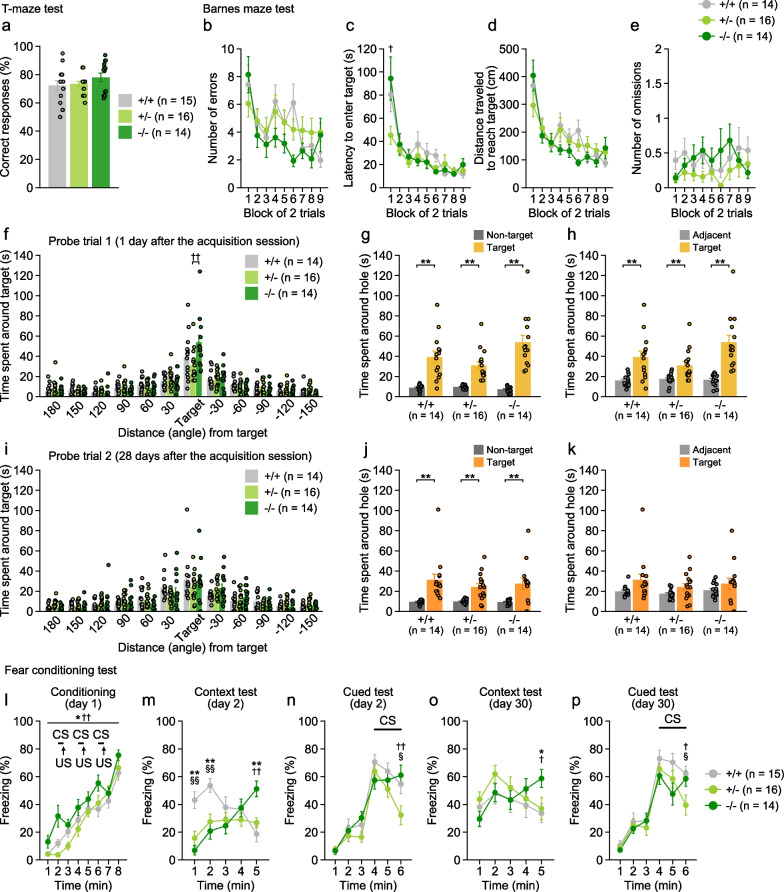
Memory functions in 5-HTT-deficient mice. **a** Correct responses (%) in the T-maze spontaneous alternation test. **b**–**k** Barnes maze test: **b** the number of errors to first reach the target hole, **c** latency to reach the target hole, **d** distance traveled to first reach the target hole, and **e** number of omissions during the acquisition session. **f**–**k** Time spent around the target hole in the probe trial 1 day (**f**) and 28 days (**i**) after the last acquisition session. In each probe trial, time spent around the target hole was compared to averaged time spent around 11 non-target holes (**g**, **j**) and averaged time spent around two holes adjacent to the target hole (**h**, **k**). **l**–**p** Contextual and cued fear conditioning test: freezing (%) in the conditioning session (**l** conditioned stimulus, CS, 55-dB white noise, 30 s; unconditioned stimulus, US, 0.3-mA footshock, 2 s) and in the context test (**m**) and cued test (**n**) one day after the conditioning. Freezing (%) was also measured in the context test (**o**) and cued test (**p**) 29 days after the conditioning. Values are means ± SEM. **a**–**f**, **i**, **l**–**p** Asterisks and daggers indicate statistically significant differences between groups (5-HTT−/− vs. 5-HTT+/+, *p < 0.05 and **p < 0.01; 5-HTT−/− vs. 5-HTT+/−, ^†^p < 0.05 and ^††^p < 0.01; 5-HTT+/− vs. 5-HTT+/+, ^§^p < 0.05 and ^§§^p < 0.01). **g**, **h**, **j** Asterisks represent statistical significance with paired t tests (**p < 0.01)

The T-maze spontaneous alternation test was performed to evaluate working memory. The percentage of correct responses in the T-maze did not differ between the three genotypes (Fig. 4a), suggesting normal working memory in 5-HTT−/− mice.

In the Barnes maze test of spatial memory, there were no significant main effects of genotype and genotype × block interactions on the number of errors, the distance traveled to first reach the target hole, and the number of omission errors during the acquisition sessions (Fig. 4b, d, e). A significant genotype × block interaction (F_16,328_ = 2.04, p = 0.0105) and a significant simple main effect of genotype were observed for latency to reach the target hole in the first block (Fig. 4c; +/− vs. −/−, p = 0.0168), and there were no genotype differences in the latency to reach the target hole from the second to the last block. In the probe test conducted one day after the last acquisition session to assess memory retention, a significant effect of genotype was found on the time spent around the target hole (Fig. 4f), and 5-HTT−/− mice spent longer time around the target hole than other genotypes, although the difference between 5-HTT−/− and 5-HTT+/+ mice did not reach significance (−/− vs. +/+, p = 0.0793; −/− vs. +/−, p = 0.0057; +/− vs. +/+, p = 0.2973). Each genotype of mouse spent more time around the target hole than the mean time spent around the non-target holes (Fig. 4g; +/+, t_13_ = 4.53, p = 0.0006; +/−, t_15_ = 5.42, p < 0.0001; −/−, t_13_ = 6.03, p < 0.0001) and mean time spent around the holes adjacent to the target (Fig. 4h; +/+, t_13_ = 3.70, p = 0.0027; +/−, t_15_ = 3.87, p = 0.0015; −/−, t_13_ = 4.59, p = 0.0005). The probe test data suggest that 5-HTT−/− mice may have an increased motivation to escape from the maze due to their heightened anxiety-like phenotype or may have an enhanced recent spatial memory. In the second probe test 28 days after the acquisition, there was no significant effect of genotype on the time spent around the target hole (Fig. 4i). In the second probe test, each genotype of mice spent longer time around the target hole than the mean time spent around the non-target holes (Fig. 4j; +/+, t_13_ = 3.50, p = 0.0039; +/−, t_15_ = 3.99, p = 0.0012; −/−, t_13_ = 3.04, p = 0.0095). There was no significant difference between the time spent around the target and adjacent holes in 5-HTT−/− mice (t_13_ = 1.04, p = 0.3169), and 5-HTT+/+ and 5-HTT+/− mice tended to spend more time around the target hole than time around the adjacent holes (Fig. 4k; +/+, t_13_ = 2.01, p = 0.0652; +/−, t_15_ = 1.99, p = 0.0653).

### Impaired fear memory in 5-HTT-deficient mice

In the conditioning session of the fear conditioning test, there were significant main effects of genotype on freezing and distance traveled (Fig. 4l and Additional file 1: Fig. S1a). A significant genotype × time interaction was found on the distance traveled (Additional file 1: Fig. S1a). 5-HTT−/− mice showed more freezing than other genotypes (vs. +/+, p = 0.0102; vs. +/−, p = 0.0030), while 5-HTT+/− and 5-HTT+/+ mice did not differ in freezing (p = 0.6683). In addition, 5-HTT−/− mice traveled a shorter distance than other genotypes at the 1st and 2nd time blocks (all p < 0.05). 5-HTT+/− mice traveled a longer distance than 5-HTT+/+ mice during the 2nd time block (p = 0.0089). Distance traveled for 4 s during and after 2-s footshocks was analyzed to assess shock sensitivity. A significant main effect of genotype was found on the distance traveled in response to the first footshock, but not to the second and third footshocks (Additional file 1: Fig. S1f–h). 5-HTT−/− traveled a shorter distance in response to the first footshock than 5-HTT+/+ and 5-HTT+/− mice (p = 0.0491 and p = 0.0016, respectively; 5-HTT+/− vs. 5-HTT+/+, p = 0.1880).

In the context test, approximately 24 h after conditioning, there were significant main effects of genotype and significant genotype × time interactions on freezing and distance traveled (Fig. 4m and Additional file 1: Fig. S1b). 5-HTT−/− and 5-HTT+/− mice showed less freezing and traveled longer distances than 5-HTT+/+ mice in the first and second blocks (all p < 0.05), while 5-HTT−/− displayed more freezing and shorter distance traveled than other genotypes in the 5th time block. In the cued test without CS presentation, there were no significant main effects of genotype and no significant interactions on freezing and distance traveled (Fig. 4n and Additional file 1: Fig. S1c). During the last 3 min with CS in the cued test, there were significant genotype × time interactions in freezing and distance traveled. In the last time block with CS, 5-HTT+/− mice showed less freezing and traveled a longer distance than other genotypes (all p < 0.05).

The context test and the cued test were conducted 29 days after conditioning to further assess remote memory. In the second trial of the context test, there was a significant genotype × time interaction on freezing but not on distance traveled (Fig. 4o and Additional file 1: Fig. S1d). During the 5th time block, 5-HTT−/− mice exhibited more freezing than 5-HTT+/+ and 5-HTT+/− mice (p = 0.0205 and p = 0.0407, respectively). In the cued test 29 days after conditioning, there were no significant main effects of genotype and no significant genotype × time interactions on freezing and distance traveled during the first 3 min without CS (Fig. 4p and Additional file 1: Fig. S1e). During the last 3 min of the cued test with CS, there were significant genotype × time interactions on freezing and distance traveled. 5-HTT−/− mice traveled a longer distance than 5-HTT+/+ mice in the 4th and 5th time block (all p < 0.05), and 5-HTT+/− mice exhibited less freezing and traveled a longer distance traveled than 5-HTT+/+ and 5-HTT−/− mice in the 6th time block (all p < 0.05).

### Altered composition of intestinal microbiome in 5-HTT-deficient mice

**Fig. 5:**
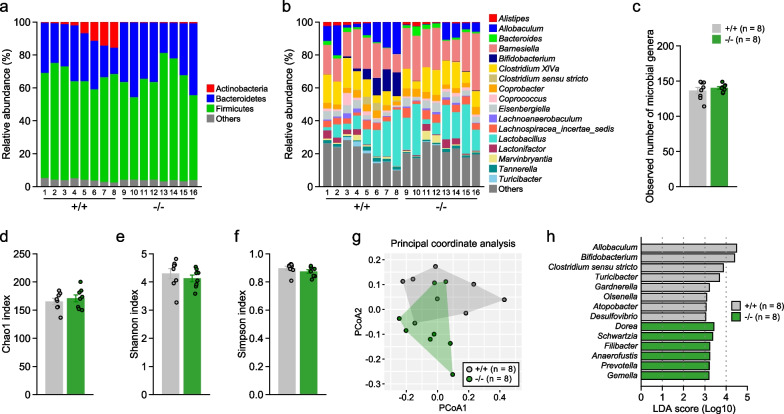
Intestinal microbial composition in 5-HTT-deficient mice. Relative abundance (%) of microbiota at the phylum level (**a**) and at the genus level (**b**) in fecal sample in each mouse (taxonomies with relative abundance of less than 1% or not identified were included in 'Others'). **c**–**f** Alpha diversity of the microbiota at the genus level: **c** observed number of microbial genera, **d** Chao1 index, **e** Shannon index, and **f** Simpson index. Values are means ± SEM. **g** Beta diversity of the microbiota at the genus level was visualized with principal coordinate analysis (PCoA) using Bray–Curtis dissimilarity. **h** Taxa differentially abundant between 5-HTT−/− and 5-HTT+/+ mice was identified by the linear discriminant analysis (LDA) effect size (LEfSe) method (LDA score > 3, p < 0.05)

The microbial taxa with a mean relative abundance greater than 1% in any group at the phylum and genus level are shown in Figs. 5a and b (taxonomies with a relative abundance less than 1% or not identified were included in 'Others'; for details, see Additional file 4: Tables S3 and S4). The most abundant taxa at the phylum level were Actinobacteria, Bacteroidetes, and Firmicutes. Statistical analysis showed that 5-HTT+/+ mice had a higher abundance of Actinobacteria than 5-HTT−/− mice (p < 0.05). Next, alpha and beta diversity were examined using data from the identified 259 taxa at genus level. For alpha diversity indices (Fig. 5c–f), there were no significant effects of genotype on observed species, Chao1 index, Shannon index, and Simpson index (p > 0.05). Beta diversity, representing differences in microbial composition, differed between genotypes when visualized with principal coordinate analysis (PCoA) using Bray–Curtis dissimilarity (Fig. 5g), and permutational multivariate analysis of variance (PERMANOVA) showed a tendency toward a difference in Bray–Curtis dissimilarity (F_1,14_ = 2.1756, p = 0.098). Further analysis using the linear discriminant analysis (LDA) effect size (LEfSe) method revealed that there were significant effects of genotype on 14 genera (Fig. 5h: LDA score > 3, p < 0.05), showing that 5-HTT−/− mice had reduced abundances of 8 genera (*Allobaculum*, *Bifidobacterium*, *Clostridium sensu stricto*, *Turicibacter*, *Gardnerella*, *Olsenella*, *Atopobacter*, *Desulfovibrio*) and increased abundances of 6 genera (*Dorea*, *Schwartzia*, *Filibacter*, *Anaerofustis*, *Prevotella*, and *Gemella*) compared with 5-HTT+/+ mice (Additional file 2: Fig. S2).

### Plasma corticosterone levels

There was no significant main effect of genotype and no significant genotype × phase interaction on plasma corticosterone levels (genotype effect, F_1,9_ = 0.50, p = 4981; interaction, F_2,18_ = 0.33, p = 0.7237). 5-HTT−/− and 5-HTT+/+ mice did not differ in corticosterone levels before exposure to the forced swim test, immediately after the test and 90 min after the test (Additional file 3: Fig. S3).

### Brain c-Fos expression

**Fig. 6:**
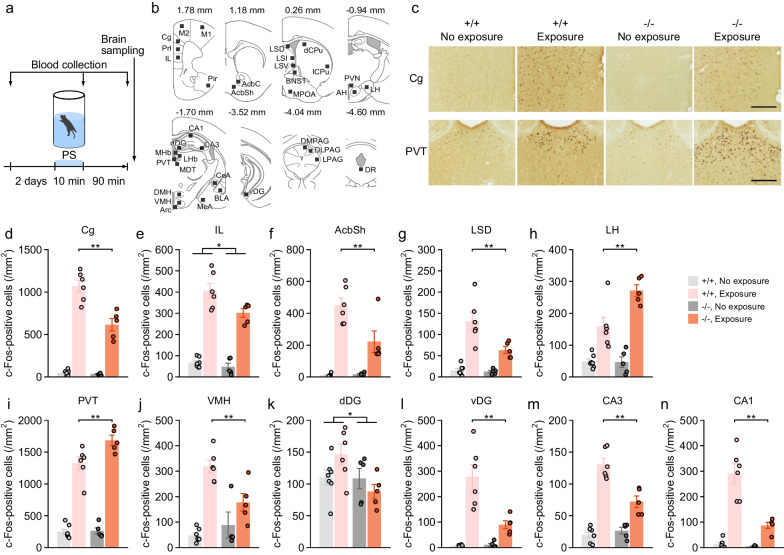
Brain c-Fos expression in 5-HTT-deficient mice. **a** Experimental design for brain sampling. 5-HTT−/− and 5-HTT+/+ mice were either exposed to the Porsolt forced swim test (PS, Exposure) or were left undisturbed until sacrifice without any exposure to behavioral testing (No exposure). **b** Schematic diagram of the brain areas in which c-Fos expression was quantified (brain images were adapted with permission from ref. [83]). **c** Representative photomicrographs of c-Fos immunoreactivity on brain regions (cingulate cortex, Cg; paraventricular thalamus, PVT). **d**–**n** Number of c-Fos-positive cells (/mm^2^) in the brain areas: (**d**) cingulate cortex (Cg), **e** infralimbic cortex (IL), **f** nucleus accumbens shell (AcbSh), **g** dorsolateral septum (LSD), **h** lateral hypothalamus (LH), **i** paraventricular thalamus (PVT), **j** ventromedial hypothalamus (VMH), **k** dorsal dentate gyrus (dDG), **l** ventral dentate gyrsu (vDG), **m** CA3, and **n** CA1. Scale bar, 200 μm. Values are means ± SEM. Asterisks indicate statistically significant differences between 5-HTT−/− and 5-HTT+/+ mice (*p < 0.05 and **p < 0.01)

To examine genotype differences in neuronal activation in brain regions associated with depression-related behavior, we performed immunohistochemistry for the protein of the immediate early gene c-fos in the brain of mice exposed or not exposed to the forced swim test (Fig. 6a–c and Additional file 4: Table S5). Two-way ANOVAs revealed significant main effects of test exposure on c-Fos expression in all brain regions examined in this study except the dorsal dentate gyrus (dDG), where c-Fos expression was increased by forced swim exposure. There were no significant main effects of genotype and no significant genotype × exposure interactions on c-Fos expressions in some brain regions (Additional file 4: Table S5), including the paraventricular nucleus of the hypothalamus (PVN) and dorsomedial hypothalamus (DMH), which are related to the regulation of circulating corticosterone levels. Significant main effects of genotype and/or significant genotype × exposure interactions were found in the cingulate cortex (Cg), primary motor cortex (M1), secondary motor cortex (M2), infralimbic cortex (IL), piriform cortex (Pir), nucleus accumbens shell (AcbSh), dorsal part of the lateral septal nucleus (LSD), lateral hypothalamus (LH), paraventricular nucleus of the thalamus (PVT), CA1, CA3, dorsal DG (dDG), ventral DG (vDG), and ventromedial hypothalamus (VMH), although genotype × exposure interactions were marginally significant in the IL and dDG (Additional file 4: Table S5). 5-HTT−/− mice showed an increased number of c-Fos-positive cells in the PVT and LH, and a decreased number of c-Fos-positive cells in the Cg, M1, M2, Pir, IL, AcbSh, LSD, CA1, CA3, dDG, vDG, and VMH, after exposure to the forced swim test, compared to 5-HTT+/+ mice (Fig. 6d–n).

## Discussion

The present study revealed the behavioral profiles of 5-HTT−/− mice with a C57BL/6JJcl genetic background. Some behavioral phenotypes, especially anxiety-like and depression-related behaviors, have been well studied in the previous studies, while the other domains of behavior have not been fully investigated; thus, our study not only replicated the previous findings that 5-HTT−/− mice show increased anxiety-like and depression-related behaviors, demonstrating that 5-HTT−/− mice serve as a reliable animal model for depression, but also, to our knowledge, revealed novel behavioral phenotypes of 5-HTT-deficient mice, such as decreased social behavior in novel and familiar environments, no obvious deficits in recent and remote spatial memory, no generalized fear response, and altered contextual fear memory. Analysis of 16S rRNA gene amplicons revealed that 5-HTT−/− mice had altered gut microbiota compared to 5-HTT+/+ mice. Brain c-Fos expression analysis showed that the number of c-Fos-positive cells was higher in the paraventricular thalamus and lateral hypothalamus and lower in the prefrontal cortical regions, nucleus accumbens shell, dorsolateral septal nucleus, hippocampal regions, and ventromedial hypothalamus after exposure to the behavioral paradigm assessing depression-related behavior in 5-HTT−/− mice than in 5-HTT+/+ mice. These data indicate that genetic deletion of 5-HTT causes alterations in various domains of behavior, gut microbial composition, and brain c-Fos expression in brain regions associated with the regulation of anxiety and depression.

### Increased body weight and possible sensory and motor dysfunction in 5-HTT−/− mice

The results of the present study for physical characteristics and sensory and motor functions are consistent with previous studies reporting that 5-HTT−/− mice showed an increased body weight [11, 16, 19], normal body temperature [18, 84], a tendency toward increased hot plate latency [18], decreased wire-hang latency [11], and decreased rotarod latency [11], compared to 5-HTT+/+ and 5-HTT+/− mice. Our study found no genotype difference in grip strength, indicating normal forepaw muscle strength in 5-HTT-deficient mice. The decreased wire-hang and rotarod latencies were observed even when their body weights were considered as a covariate. These findings suggest two possibilities: one possibility is that 5-HTT−/− mice had lower whole-body muscle strength and reduced motor function, and the other possibility is that the reduced latencies to fall off may reflect a depressive state [85, 86]. Mice homozygous deficient in 5-HTT showed reduced startle responses to loud noises compared to 5-HTT+/+ mice, consistent with a recent study ([29]; but see ref. [18, 19]). The altered startle responses would not be due to loss of auditory function because the normal hearing was observed in 5-HTT−/− mice [29], although the possibility that their decreased motor functions and hypoactive phenotype lead to the reduced startle responses could not be excluded. Our data suggest that 5-HTT+/− mice have normal physical, sensory, and motor functions.

### Increased anxiety-like and depression-related behaviors in 5-HTT−/− mice

The present study confirmed that 5-HTT−/− mice showed increased anxiety-like behaviors in different types of tests, including the open field, light/dark transition, and elevated plus maze tests, as reported in previous studies (see Additional file 4: Table S6). An early study reported that depression-related phenotypes in 5-HTT KO mice were dependent on the genetic background [11]. 5-HTT−/− mice on the 129S6 background exhibited increased immobility in the forced swim test and decreased immobility in the tail suspension test [11, 14], whereas 5-HTT−/− mice on the C57BL/6 J background showed no alternation in immobility time in the forced swim and tail suspension tests [11]. Our data showed that 5-HTT−/− mice on the C57BL/6JJcl background exhibited increased immobility in the forced swim test. There are several factors influencing behavior in the forced swim test, such as test conditions and prior test experience [54, 87]. For example, repeated exposure to the forced swim test resulted in greater immobility in 5-HTT−/− mice on the C57BL/6 J background than in wild-type mice [25, 31]. In the present study, previous testing experience across the battery of behavioral tests might have led to heightened depression-related behavior in 5-HTT−/− mice, even in the first trial. In addition, 5-HTT−/− mice showed an increase in immobility from the second minute of the test compared to 5-HTT+/+ mice on the first day of the forced swim test, and the immobility in all three genotypes of mice reached a plateau after the sixth minute of the test. These results indicate that immobility increased earlier in 5-HTT−/− mice than in 5-HTT+/+ mice. The second test confirmed the finding of increased immobility observed in 5-HTT−/− mice on the first day of testing. Immobility in the first minute of the second test was higher than in the first test, suggesting that a learned fear to the unavoidable situation was normal in 5-HTT−/− mice. The tail suspension test is conceptually similar to but methodologically different from the forced swim test for assessing depression-related behavior. A previous study showed that exposure to the tail suspension test did not induce changes in brain monoamine concentrations, whereas the forced swim test did, suggesting that the two tests involve different neural mechanisms [88]. In our previous studies, the results of genotype difference in immobility observed in the forced swim test were not necessarily consistent with those found in the tail suspension test in some strains of mutant mice [89–94], and it is not rare that the two tests yield seemingly inconsistent results. The exact reason why significant differences in immobility were observed between 5-HTT−/− and 5-HTT+/+ mice in the forced swim test but not in the tail suspension test remains unclear. Therefore, further study is needed to understand the mechanisms underlying the link between 5-HTT deficiency and immobility in the tests. 5-HTT−/− and 5-HTT+/− mice consumed less sucrose solution and showed a lower sucrose preference than 5-HTT+/+ mice, although there was no significant difference in sucrose preference between 5-HTT−/− and 5-HTT+/+ mice, which is partially consistent with the previous study in mice and rats [22, 95]. These results suggest a trend toward an anhedonia-like phenotype in 5-HTT-deficient mice. Although 5-HTT−/− mice showed a marked decrease in distance traveled in novel environments such as the open field, there were no genotype differences in locomotor activity in a home cage in a familiar environment during the light phase when other behavioral tests were performed. It is suggested that the hypoactive phenotype is specific to novel environments. Thus, increased anxiety-like and depression-related behaviors could not be explained solely by changes in motor function and general activity. The present study also indicates that locomotor activity and anxiety-like and depression-related behaviors of 5-HTT+/− mice do not differ from those of 5-HTT+/+ mice.

### Altered social behavior in 5-HTT-deficient mice

In the social interaction test, which has been used to assess anxiety in rodents [96], the decreased distance traveled in 5-HTT−/− mice was observed, possibly resulting in decreased social contact. Our results suggest an increased anxiety-like phenotype in a social situation in 5-HTT−/− mice. Interestingly, 5-HTT−/− mice spent more time in physical contact with a novel conspecific than 5-HTT+/+ mice. Similar results for increased social contacts in 5-HTT−/− mice were observed at the beginning of the home cage social interaction test. These behavioral outcomes in a novel environment in 5-HTT−/− mice may be partly due to their hypoactive phenotype. Together with the reduced number of social contacts in the reciprocal social interaction test, the results of the three-chamber paradigm in the present study confirmed the reduced social preference in 5-HTT−/− and 5-HTT+/− mice, as shown in the previous study [28]. These findings suggest that 5-HTT deficiency result in reduced social behavior.

### No apparent deficits in working and spatial memory and altered fear memory in 5-HTT−/− mice

Enhanced spatial working memory, as indicated by increased spontaneous alternation behavior in the T-maze task, was reported in 5-HTT−/− mice [27]. A similar trend for genotype effect on spontaneous alternation was observed in the present study, although our results did not reach statistical significance. The inconsistent results may be due to methodological differences between the studies, such as testing procedures and genetic background. The procedure used in the previous study consisted of a forced-choice run followed by 14 consecutive free-choice trials [27], in which there would be inter-trial interference due to the continuous nature of the task, similar to the Y-maze task, potentially increasing cognitive demands [97]. In our study, each trial consisted of one forced and one free choice run with an inter-trial interval of 60 s, which is expected to reduce the inter-trial interference and the task difficulty. The genetic background of the animals was C57BL/6N background in the previous study [27] and C57BL/6JJcl background in our study. C57BL/6N showed fewer different arm choices in the first eight arm entries, which is indicative of decreased working memory, compared to C57BL/6J in the eight-arm radial maze test [98]. The lower basal level (approximately 50% chance level) of spontaneous alternation observed in 5-HTT+/+ mice with C57BL/6N background [27] might allow for detecting the possible improving effect of 5-HTT deficiency on working memory. As evidenced by previous studies on anxiety-like and depression-related behaviors [11, 12], our findings also highlight the importance of considering genetic background when studying learning and memory in 5-HTT-deficient mice. In the Barnes maze test, 5-HTT−/− mice spent slightly more time around the target hole in the probe trial one day after the acquisition session, but showed no difference in time in the target 28 days after the acquisition compared to 5-HTT+/+ mice. The Barnes maze test is based on the innate motivation of rodents to avoid the aversive open and brightly lit environment. Thus, although the results suggest that 5-HTT deficiency may contribute to an improvement in recent spatial memory as seen in the T-maze test, the increased time spent in the target area in 5-HTT−/− mice could be explained by an increased motivation to escape the maze due to an enhanced anxiety-like phenotype.

Decreased freezing and increased distance traveled were observed in 5-HTT−/− and 5-HTT+/− mice during the first 2 min of the context test, suggesting impaired contextual fear memory induced by 5-HTT deficiency. Interestingly, 5-HTT−/− mice exhibited increased freezing during the last minute of the context test. The time-dependent changes in freezing might reflect an initial increase in the flight and escape response due to their heightened fear and subsequently their hypoactive phenotype or delayed fear memory retrieval. In the cued test with altered context one day after conditioning, the present study showed no genotype differences in overall freezing during no CS presentation and CS presentation, consistent with previous studies [31, 32]. These results suggest that 5-HTT may not be involved in the control of generalized fear and recent cued fear memory. However, the reason why 5-HTT+/− mice showed reduced freezing during the last minute of the test remains unclear. In the retest 29 days after conditioning, 5-HTT−/− mice also showed higher levels of freezing during the last minute of the context test, suggesting that deletion of 5-HTT gene does not interfere with retrieval of remote contextual fear memory.

### 5-HTT deficiency-induced alteration in intestinal microbial composition

Accumulating evidence suggests that neuropsychiatric disorders, including bipolar disorder and major depression, are associated with an altered gut microbiota composition in preclinical and clinical studies [99–101]. There is a recent report on the gut microbial composition in 5-HTT−/− mice, which showed higher abundances of Bacilli species, including the genera Lactobacillus, Streptococcus, Enterococcus and Listeria, and lower abundances of Bifidobacterium species and *Akkermansia muciniphilia* than 5-HTT+/+ mice [43]. The present study showed that 5-HTT−/− mice had higher abundance of Streptococcus and lower abundance of Bifidobacterium than 5-HTT+/+ mice, replicating the key findings of the previous report for altered gut microbial composition in 5-HTT−/− mice [43]. Our study also identified altered abundances of some other microbial genera which were not reported in the previous study [43], showing that 5-HTT−/− mice had decreased abundances of Allobaculum and Clostridium sensu stricto in fecal samples. Depression and obesity tend to co-occur in individuals [102]. Allobaculum and Clostridium sensu stricto are negatively associated with inflammation, insulin resistance, lipid metabolism, and obesity [103–107]. Such microbial composition may explain increased depressive and obesity phenotypes such as increased body weight, possible insulin resistance, and hyperglycemia in 5-HTT−/− mice [108]. In this study, decreased abundance of Bifidobacterium and Turicibacter and increased abundance of Prevotella were also observed in 5-HTT−/− mice. Similar changes in relative abundances of Bifidobacterium, Turicibacter, and Prevotella have been reported in human patients with depression compared to healthy controls [37, 109–111], while there are inconsistencies in the gut microbial compositions between human studies of depression [100, 112]. The present findings strengthen the notion that 5-HTT−/− mice with altered gut microbial composition can be used as an animal model of depression.

### Possible brain regions involved in the depressive state induced by 5-HTT deficiency

Stress can trigger the hypothalamus–pituitary–adrenal (HPA) axis response through the activation of neurons in some brain regions, including the paraventricular nucleus of the hypothalamus (PVN) and dorsomedial hypothalamus (DMH), resulting in elevations in circulating corticosterone [113]. Our results of brain c-Fos expression suggest that PVN and DMH neurons may be activated at a similar level between 5-HTT−/− and 5-HTT+/+ mice, which seems to consistent with the results of this study and a previous study showing that no differences in plasma corticosterone levels after exposure to the forced swim test as well as immobilization stress were observed in the two genotypes of mice [114]. These findings suggest a normal endocrine response involving the adrenal glands after stress exposure in 5-HTT−/− mice, although some reports showed that 5-HTT−/− mice have reduced basal corticosterone and increased adrenocorticotropic hormone [115, 116].

Brain mapping of neuronal activation by c-Fos expression in response to the forced swim test may allow us to understand the neural circuit involved in the induction and facilitation of the depressive state induced by 5-HTT deficiency. The present results of c-Fos expression analysis suggest hypoactivation of the PFC (cingulate, infralimbic, piriform, and motor cortex), nucleus accumbens shell, dorsolateral septum, ventromedial hypothalamus, and hippocampus (CA1, CA3, and dentate gyrus) and hyperactivation of the paraventricular thalamus (PVT) and lateral hypothalamus (LH) in 5-HTT−/− mice. Functional brain imaging studies of human patients with depression have reported that the PFC (including the anterior cingulate cortex and ventromedial prefrontal cortex), striatum, hippocampus, and amygdala have important implications for the neurobiological mechanisms of depression and anxiety [117, 118]. The rodent homolog of the ventromedial prefrontal cortex is the infralimbic cortex [119], which sends a dense projection to the nucleus accumbens shell [120, 121]. The infralimbic cortex and its projection to the nucleus accumbens shell have been reported to play a role in affective processing and the suppressing learned negative emotional states [122, 123]. The ventromedial hypothalamus and the septo-hippocampal circuit are involved in escape and defensive responses, mood, and motivation [124–129]. The PVT participates in the control of emotional behavior, with its projections to the PFC and amygdala promoting anxiety-like and depression-related behaviors and fear memory [130–134], and to the accumbens inhibiting anxiety-like behavior ([135, 136]; for reviews, see ref. [137]). Activation of the LH bidirectionally modulates anxiety-like behavior and feeding via GABAergic and orexin neurons [138, 139]. Given the functional interactions between these brain structures, altered neuronal activation in the brain regions in 5-HTT−/− mice may be characteristic of the increased depression-related behavior observed in the forced swim test. However, there are limitations in considering c-Fos expression as a marker of neuronal activity [140, 141], and therefore it needs to be further confirmed whether c-Fos expression truly reflects the differences in neuronal activity during the forced swim test between 5-HTT−/− and 5-HTT+/+ mice. For example, alpha-CaMKII heterozygous knockout mice have an immature dentate gyrus (iDG) phenotype, in which the DG neurons are in a pseudo-immature status [142, 143]. The mutants showed dramatically reduced c-Fos and Arc expressions in the brain after electric footshock or a memory task compared to wild-type mice [142, 144], while an in vivo calcium imaging study showed that the estimated firing rate from calcium signals in the DG neurons did not differ between the mutants and wild-type mice [145]. Thus, c-Fos immunohistochemistry has low temporal resolution but may be useful to identify brain regions activated by exposure to the behavioral test. Another limitation is that the present study could not exclude the possibility of the influence of stress on c-Fos expression by blood collection after the forced swim test.

### 5-HT hypothesis of depression

The 5-HT hypothesis of depression is one of the widely studied biological hypotheses that depression is caused by reduced 5-HT activity or concentration. Recent meta-analytic studies and a systematic umbrella review provide no consistent evidence of an association between 5-HT, 5-HTT gene polymorphism, and depression and no support for the hypothesis [146]. On the other hand, some meta-analytic studies showed weak and inconsistent evidence of reduced 5-HTT binding in some brain areas [147–149], which would lead to an increased possibility of synaptic availability of 5-HT, in people with depression [146]. 5-HTT−/− mice had increased levels of extracellular 5-HT [150, 151] and decreased tissue concentrations of 5-HT and its metabolite 5-HIAA [152] in the brain compared to 5-HTT+/+ mice. Thus, 5-HTT−/− mice may resemble humans with a subtype of depression with serotonergic dysregulation. Anxiety and depression are a co-occurring symptom in individuals with autism spectrum disorder (ASD) with a core symptom of social deficits [153, 154]. ASD has been reported to be associated with elevated blood 5-HT levels and reduced brain 5-HT/5-HTT binding [155–159]. A previous study [28] and the present findings indicate that 5-HTT deficient mice may also be a useful animal model for studying ASD with anxiety and depression.

## Conclusions

The present study shows that 5-HTT−/− mice exhibit various behavioral phenotypes, such as decreased locomotor activity, heightened anxiety-like and depression-related behaviors, decreased social behavior in novel environments, no deficits in recent and remote spatial memory, normal generalized fear response and cued fear memory, and altered contextual fear memory. The behavioral results of previous studies [11, 14, 18, 19, 24, 25] and the present study indicate that significant increases in anxiety-like and depression-related behaviors are a robust, well-replicated behavioral phenotype in 5-HTT−/− mice. These findings reveal the role of 5-HTT on brain function and strengthen the notion that 5-HTT-deficient mice have a high face and construct validity of the animal model for studying anxiety and depression with altered serotonergic function. This study also showed 5-HTT deficiency-induced changes in the abundance of some microbial genera in feces, offering novel targets for further research on the role of gut microbiota in behavioral changes induced by 5-HTT deficiency. Furthermore, brain c-Fos analysis helps to understand the brain circuits and neuronal activation associated depressive state induced by 5-HTT deficiency. Our results suggest a potential link between 5-HTT deficiency-induced changes in gut microbiota, brain neuronal activation, and behavior, although further research is needed to elucidate the causal relationship between the gut-brain-behavior. Taken together, the results of the present study highlight the importance of 5-HTT in regulating brain function, possibly in part via the gut-brain axis, and also provide useful insights into the understanding of neuronal and circuitry mechanisms underlying brain dysfunction caused by dysregulated serotonin neurotransmission.

## Supplementary Information

**Additional file 1: Figure S1.** Distance traveled in fear conditioning test in 5-HTT-deficient mice. (a–h) Contextual and cued fear conditioning test: distance traveled (cm) in the conditioning session (a: conditioned stimulus, CS, 55-dB white noise, 30 s; unconditioned stimulus, US, 0.3-mA footshock, 2 s) and in the context test (b) and cued test (c) one day after the conditioning. Distance traveled (cm) was also measured in the context test (d) and cued test (e) 29 days after the conditioning. In the conditioning session, to assess footshock sensitivity, distance traveled (cm) was measured from images recorded at high frame rate for 6 s from 2 s before electric footshock (2-s period) to 2 s after exposure to footshock. Values are means ± SEM. (a–f, i, l–p) Asterisks and daggers indicate statistically significant differences between groups (5-HTT−/− vs. 5-HTT+/+, * p < 0.05 and ** p < 0.01; 5-HTT−/− vs. 5-HTT+/−, † p < 0.05 and †† p < 0.01; 5-HTT+/− vs. 5-HTT+/+, § p < 0.05 and §§ p < 0.01).

**Additional file 2: Figure S2.** Relative abundance of intestinal microbiota at the genus level in 5-HTT-deficient mice. (a–n) Relative abundance (%) of microbiota at the genus level in fecal samples of 5-HTT−/− and 5-HTT+/+ mice. Taxa differentially abundant between 5-HTT−/− and 5-HTT+/+ mice, which was identified by the linear discriminant analysis (LDA) effect size (LEfSe) method (LDA score > 3, p < 0.05). (a) Allobaculum, (b) Bifidobacterium, (c) Clostridium sensu stricto, (d) Turicibacter, (e) Gardnerella, (f) Olsenella, (g) Atopobacter, (h) Desulfovibrio, (i) Dorea, (j) Schwartzia, (k) Filibacter, (l) Anaerofustis, (m) Prevotella, and (n) Gemella. Values are means ± SEM. Asterisks indicate statistically significant differences between groups (* p < 0.05, ** p < 0.01, and *** p < 0.001).

**Additional file 3: Figure S3.** Plasma corticosterone levels in 5-HTT-deficient mice. Blood were collected from 5-HTT−/− and 5-HTT+/+ mice 2 days before the Porsolt forced swim test (PS), immediately after the PS test, and 90 min after the PS test. Corticosterone levels (ng/mL) in the blood samples were measured. Values are means ± SEM.

**Additional file 4: Table S1.** A battery of behavioral tests in 5-HTT-deficient and wild-type mice. **Table S2.** Statistical analysis of behavioral data of 5-HTT-deficient and wild-type mice. **Table S3.** Relative abundance of intestinal microbiota at the phylum level in 5-HTT−/− and 5-HTT+/+ mice. **Table S4.** Relative abundance of intestinal microbiota at the genus level in 5-HTT−/− and 5-HTT+/+ mice. **Table S5.** Number of c-Fos-positive cells in the brain in 5-HTT−/− and 5-HTT+/+ mice. **Table S6.** Behavioral study of 5-HTT-deficient animals

## Data Availability

The data used in the study are available from the corresponding author on reasonable request.
