# Supplementary figures and images for: Behavioral phenotype, intestinal microbiome, and brain neuronal activity of male serotonin transporter knockout mice
Source: Mol Brain. 2023 Mar 29;16:32. doi: 10.1186/s13041-023-01020-2 (PMC10061809; doi:10.1186/s13041-023-01020-2)

# Fear conditioning test

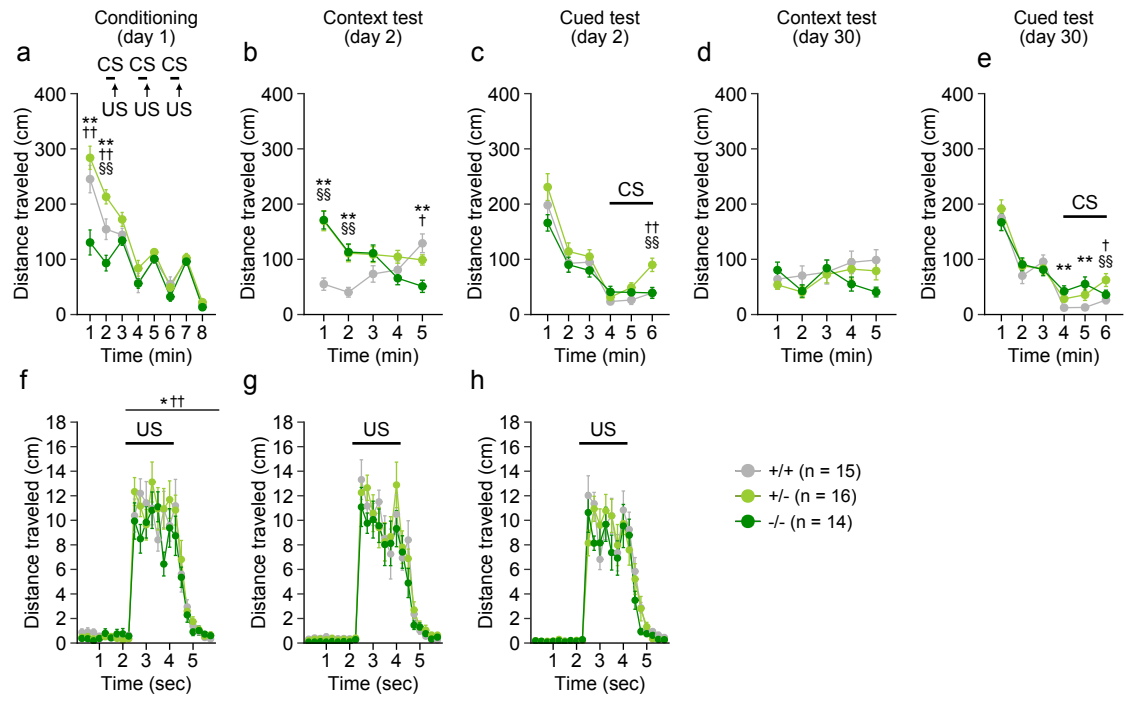

Supplement: Supplementary file 1 — Additional file 1: Figure S1. Distance traveled in fear conditioning test in 5-HTT-deficient mice. (a–h) Contextual and cued fear conditioning test: distance traveled (cm) in the conditioning session (a: conditioned stimulus, CS, 55-dB white noise, 30 s; unconditioned stimulus, US, 0.3-mA footshock, 2 s) and in the context test (b) and cued test (c) one day after the conditioning. Distance traveled (cm) was also measured in the context test (d) and cued test (e) 29 days after the conditioning. In the conditioning session, to assess footshock sensitivity, distance traveled (cm) was measured from images recorded at high frame rate for 6 s from 2 s before electric footshock (2-s period) to 2 s after exposure to footshock. Values are means ± SEM. (a–f, i, l–p) Asterisks and daggers indicate statistically significant differences between groups (5-HTT−/− vs. 5-HTT+/+, * p < 0.05 and ** p < 0.01; 5-HTT−/− vs. 5-HTT+/−, † p < 0.05 and †† p < 0.01; 5-HTT+/− vs. 5-HTT+/+, § p < 0.05 and §§ p < 0.01). [file 13041_2023_1020_MOESM1_ESM.pdf]

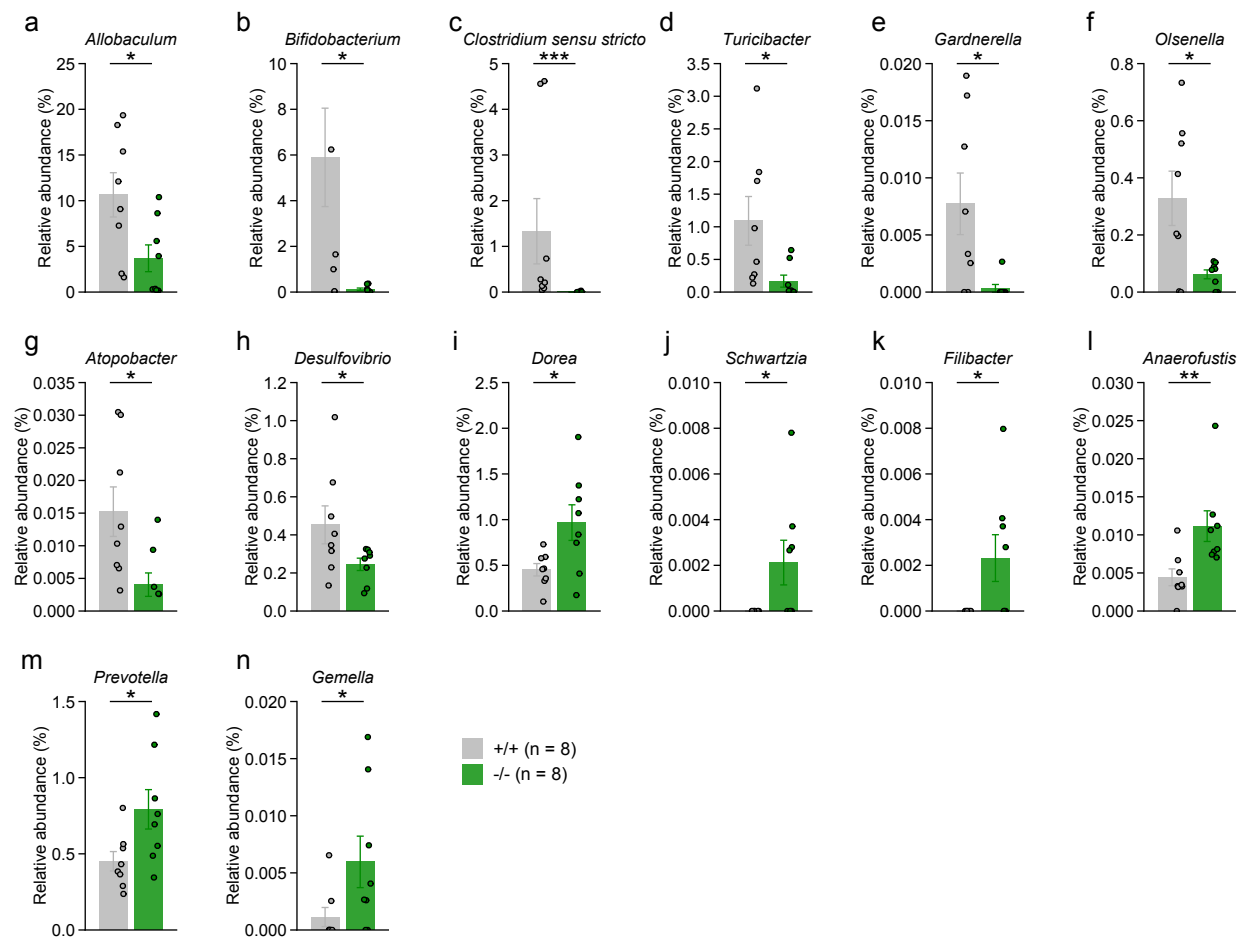

Supplement: Supplementary file 2 — Additional file 2: Figure S2. Relative abundance of intestinal microbiota at the genus level in 5-HTT-deficient mice. (a–n) Relative abundance (%) of microbiota at the genus level in fecal samples of 5-HTT−/− and 5-HTT+/+ mice. Taxa differentially abundant between 5-HTT−/− and 5-HTT+/+ mice, which was identified by the linear discriminant analysis (LDA) effect size (LEfSe) method (LDA score > 3, p < 0.05). (a) Allobaculum, (b) Bifidobacterium, (c) Clostridium sensu stricto, (d) Turicibacter, (e) Gardnerella, (f) Olsenella, (g) Atopobacter, (h) Desulfovibrio, (i) Dorea, (j) Schwartzia, (k) Filibacter, (l) Anaerofustis, (m) Prevotella, and (n) Gemella. Values are means ± SEM. Asterisks indicate statistically significant differences between groups (* p < 0.05, ** p < 0.01, and *** p < 0.001). [file 13041_2023_1020_MOESM2_ESM.pdf]

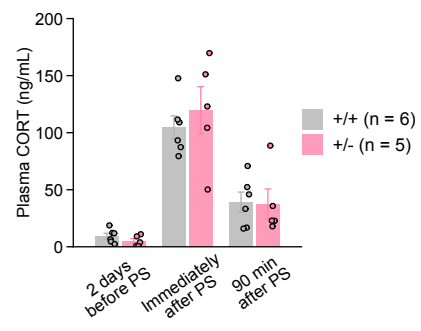

Supplement: Supplementary file 3 — Additional file 3: Figure S3. Plasma corticosterone levels in 5-HTT-deficient mice. Blood were collected from 5-HTT−/− and 5-HTT+/+ mice 2 days before the Porsolt forced swim test (PS), immediately after the PS test, and 90 min after the PS test. Corticosterone levels (ng/mL) in the blood samples were measured. Values are means ± SEM. [file 13041_2023_1020_MOESM3_ESM.pdf]
